# Engagement of Siglec-7 Receptor Induces a Pro-Inflammatory Response Selectively in Monocytes

**DOI:** 10.1371/journal.pone.0045821

**Published:** 2012-09-28

**Authors:** Stefania Varchetta, Enrico Brunetta, Alessandra Roberto, Joanna Mikulak, Kelly L. Hudspeth, Mario U. Mondelli, Domenico Mavilio

**Affiliations:** 1 Department of Infectious Diseases, Research Laboratories, IRCCS, Fondazione San Matteo and University of Pavia, Pavia, Italy; 2 Unit of Clinical and Experimental Immunology, Humanitas Clinical and Research Center, Rozzano, Milan, Italy; 3 Laboratory of Clinical and Experimental Immunology, IRCCS, Istituto Clinico Humanitas, Rozzano, Milano, Italy; University Medical Center Freiburg, Germany

## Abstract

Sialic acid binding immunoglobulin-like lectin-7 (Siglec-7) is a trans-membrane receptor carrying immunoreceptor tyrosine based inhibitory motifs (ITIMs) and delivering inhibitory signals upon ligation with sialylated glycans. This inhibitory function can be also targeted by several pathogens that have evolved to express sialic acids on their surface to escape host immune responses. Here, we demonstrate that cross-linking of Siglec-7 by a specific monoclonal antibody (mAb) induces a remarkably high production of IL-6, IL-1α, CCL4/MIP-1β, IL-8 and TNF-α. Among the three immune cell subsets known to constitutively express Siglec-7, the production of these pro-inflammatory cytokines and chemokines selectively occurs in monocytes and not in Natural Killer or T lymphocytes. This Siglec-7-mediated activating function is associated with the phosphorylation of the extracellular signal-regulated kinase (ERK) pathway. The present study also shows that sialic acid-free *Zymosan* yeast particles are able to bind Siglec-7 on monocytes and that this interaction mimics the ability of the anti Siglec-7 mAb to induce the production of pro-inflammatory mediators. Indeed, blocking or silencing Siglec-7 in primary monocytes greatly reduced the production of inflammatory cytokines and chemokines in response to *Zymosan*, thus confirming that Siglec-7 participates in generating a monocyte-mediated inflammatory outcome following pathogen recognition. The presence of an activating form of Siglec-7 in monocytes provides the host with a new and alternative mechanism to encounter pathogens not expressing sialylated glycans.

## Introduction

Sialic acid-binding immunoglobulin-like lectins (Siglecs) are a family of trans-membrane proteins generally transmitting inhibitory signals to immune cells upon ligation with their natural ligands, the sialylated carbohydrates [1], [2]. Several pathogenic microorganisms including *Neisseria meningitidis*, *Group B streptococci*, *Trypanozoma cruzi* have evolved to synthesize or capture sialic acids and incorporate these into their own glycoconjugates [3], [4], [5], [6], [7]. In this regard, there is a general agreement about the fact that pathogens learnt to express sialic acids on their surfaces to evade the hosts’ innate immune responses by targeting the inhibitory functions of Siglecs. Indeed, syialylation of glycoconjugates in pathogens appears to be crucial for pathogen survival, possibly serving as molecular mimics of host immune cell surface to avoid immune attack. Recently, a novel working hypothesis has emerged and it suggests that Siglecs have also evolved in response to the manipulation of immune responses by pathogens to provide the host with additional new activating pathways to fight pathogenic microorganisms [2], [5], [8], [9].

Siglec-7 (CDw328), a type 1 trans-membrane protein first cloned in 1999 and belonging to the human CD33-related Siglec receptors, is characterized by a sialic acid binding N-terminal V-set Ig domain, two C2-set Ig domains and an intracytoplasmic region containing one immune-receptor tyrosine based inhibitory motif (ITIM) and one ITIM-like motif. Siglec-7, also termed p75/AIRM1, is constitutively expressed on natural killer (NK) cells, monocytes and on a small subset of CD8+/CD3+ T cells. The extracellular domain of this receptor preferentially binds a (2,8)-linked disialic acids and branched α 2,6-sialyl residues, such as those displayed by ganglioside GD3 [10], [11], [12]. Similar to the other members of its family, the Siglec-7 binding site is generally masked at the cellular surface due to c*is* interactions with abundantly expressed low affinity sialic acids. Unmasking can occur after cellular activation or sialidase treatment, which cleaves the *cis* interacting low affinity ligands and makes it possible for free interactions *in trans* with highly glycosylated ligands. Even when Siglecs are masked by *cis* interactions, *trans* interactions might occur during encounters with other cells or pathogens expressing higher affinity ligands competing with *cis* [1], [13]. As for interactions with pathogens, Siglec-7 has been shown to bind *Pseudomonas aeruginosa* and *Campylobacter jejuni*, although neither the signaling pathway(s) involved in these recognitions nor potential mechanism(s) of endocytosis have been disclosed [14], [15]. We previously reported that the surface expression of Siglec-7 on NK cells is remarkably decreased in HIV-1 infected patients with high levels of ongoing viral replication, thus suggesting that a direct binding between Siglec-7 and HIV-1 might occur as it has been demonstrated for Siglec-1 [16], [17].

The inflammatory response mediated by monocytes following pathogen recognition is usually triggered by different Pathogen Recognition Receptors (PRRs), which are naturally able to recognize highly conserved molecules on microbes. Toll like receptors (TLRs) represent the best-known PRRs, but others exist. Indeed, surface receptors like mannose receptor, dectin-1, the cytosolic receptors nucleotide-binding oligomerization domain-like receptor family (NLR) and retinoic acid inducible gene I (RIG-I) belong to the family of PRRs and are involved in pathogen recognition. In this context, several studies have demonstrated that pathogens can interact simultaneously with many PRRs and the subsequent immune responses are likely dependent on all downstream signals delivered by the engaged receptors [18], [19], [20]. Although Siglec-7 is a lectin-type receptor that shares with several PRRs a similar molecular structure and a clear ability to bind pathogens, it has been shown to strongly inhibit NK cell cytotoxicity and to negatively regulate T cell receptor signaling [10], [21]. However, the functional outcome of Siglec-7 in monocytes and following pathogen recognition is still largely unclear and remains to be determined.

Here, we provide evidence that the engagement of Siglec-7 induces a selective inflammatory response in primary human monocytes. We show that cross-linking of Siglec-7 by a specific monoclonal antibody (mAb) on total peripheral blood mononuclear cells (PBMCs) triggers a remarkably high production of several pro-inflammatory cytokines and chemokines exclusively within the monocyte compartment. Blocking or silencing Siglec-7 in primary monocytes incubated with *Zymosan* yeast particles greatly reduces the production of inflammatory mediators, thus confirming that Siglec-7 participates in generating a monocyte-mediated inflammatory outcome upon pathogen recognition in the absence of a sialic-acid microenvironment.

## Results

### Ligation of Siglec-7 Induces the Production of Pro-inflammatory Molecules Selectively in Monocytes

**Figure 1:**
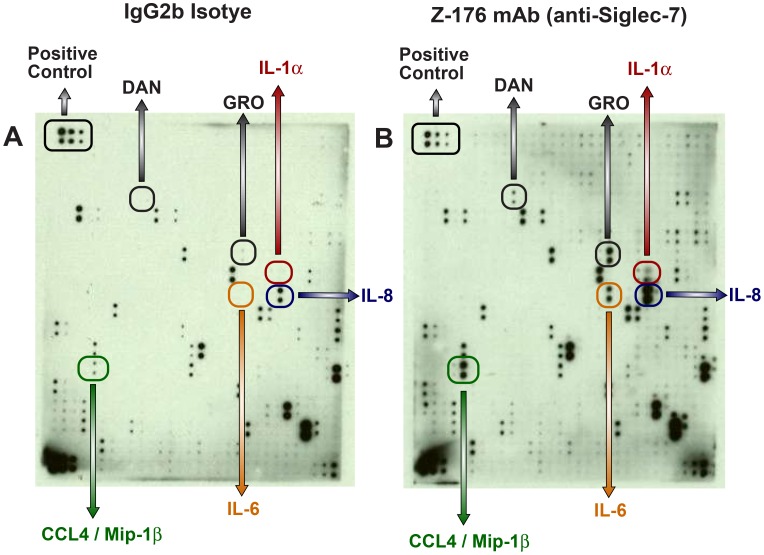
Detection of cytokines in PBMC supernatant upon engagement of Siglec-7. Secretion of IL-6, IL-1α, IL-8, CCL4/MIP-1β, GRO and DAN in the supernatant of PBMCs stimulated with the anti-Siglec-7 mAb (right panel) compared with that of PBMCs incubated with a matched IgG2b isotype control (left panel). The culture medium was collected and analysed using a semi-quantitative protein array detecting simultaneously 507 human soluble proteins. Data shown in this figure are representative of 3 independent experiments.

To evaluate Siglec-7 functions, we incubated total PBMCs in 96 flat-bottom well plates coated with Z-176, an IgG2b mAb specifically directed against Siglec-7. After 18 hours of incubation at 37°C, we collected cell supernatant and performed a semi-quantitative protein array detecting 507 different soluble proteins. Among the several molecules that showed to be differently modulated, we observed that the cross-linking of Siglec-7 in PBMCs triggered the production of IL-6, IL-1α and induced higher amounts of IL-8 and CCL4/MIP-1β compared to PBMCs incubated either with the IgG2b matched (Figure 1) or with the IgG1 unmatched (data not shown) isotype controls. There are also other molecules, such as Growth Related Oncogen (GRO) and a deadenylating nuclease (DAN), whose secretion was induced by the engagement of Siglec-7. GRO belongs to a family of chemokines specialized for monocyte arrest from flow [22], while DAN 8 also known as poly(A)-specific ribonuclease (PARN) or polyadenylate-specific ribonuclease) is the founding member of the transforming grow factor beta antagonists [23].

**Figure 2:**
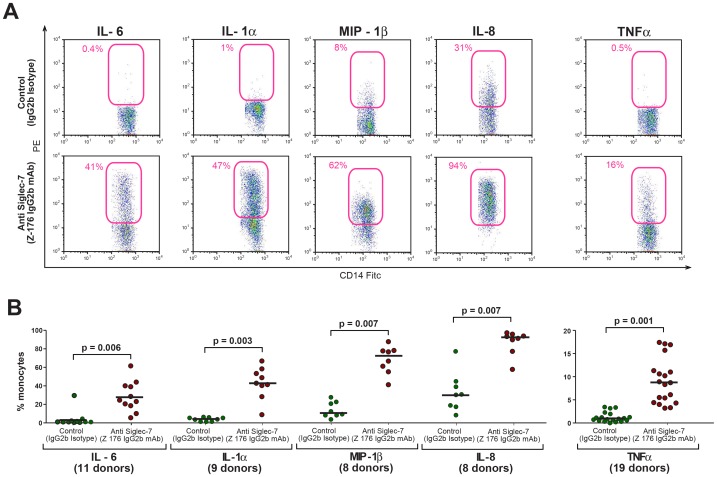
Intracellular production of pro-inflammatory cytokines and chemokines in monocytes upon engagement of Siglec-7. (A) Representative flow cytometry dot plot graphs showing the percentage of CD14^pos^ monocytes, within total PBMCs, producing IL-6, IL-1α, CCL4/MIP-1β, IL-8 and TNF-α after incubation with either the anti-Siglec-7 mAb (lower line) or the matched IgG2b isotype control (upper line). (B) Statistical summary graphs of dot plots with medians (horizontal black bars) and p values showing the percentage of CD14^pos^ monocytes producing IL-6, IL-1α, MIP-1β, IL-8 and TNF-α in response to either the anti-Siglec-7 mAb (red circles) or the matched IgG2b isotype control (green circles).

To confirm these data and to also identify the cellular compartments involved in the production of most relevant cytokines and chemokines upon Siglec-7 engagement, we performed a multicolor flow cytometry experimental approach detecting the intracellular levels of the above-mentioned inflammatory molecules within immune cells constitutively expressing Siglec-7: CD3^neg^/CD19^neg^/CD56^pos^ NK cells, CD3^pos^/CD8^pos^ T cytotoxic cells and CD14^pos^ monocytes (Figure S1) [1], [10], [11], [16]. We could not detect any NK cell- or T cell-mediated production of IL-6 upon cross-linking of Siglec-7, thus indicating that neither CD56^bright^ and CD56^dim^ NK cell subsets nor CD3^pos^/CD8^pos^ T cells are accountable for the Siglec-7-induced synthesis of this cytokine. In contrast, we found that the engagement of Siglec-7 on CD14^pos^ monocyte triggered the production of high levels of IL-6, while the intracellular levels of IL-6 produced by monocytes in the presence of both IgG2a matched and IgG1unmatched (data not shown) isotype controls were undetectable (Figures 2 A–B and S1). In line with the data illustrated in Figure 1, our results also show that the ligation of Siglec-7 with its specific mAb induced selectively in monocytes the production of IL-1α and increased significantly the production of CCL4/MIP-1β and IL-8 over isotype controls (Figure 2 A–B). Moreover, we measured the intracellular levels of other important pro-inflammatory cytokines not included in the 507-protein array: TNF-α, IL1-β and IFN-γ. We found that the cross-linking of Siglec-7, but not the ones given by either matched (Figure 2 A–B) or unmatched (data not shown) isotype-controls, triggered the production of TNF-α selectively in monocytes. We did not detect any intracellular levels of IL1-β and IFN-γ in any of the immune cells constitutively expressing Siglec-7 (data not shown). Similar to IL-6, we did not observe any Siglec-7-induced production of IL-1α, MIP-1β, IL-8 and TNF-α by NK and T cells upon cross-linking of Siglec-7 (data not shown).

**Figure 3:**
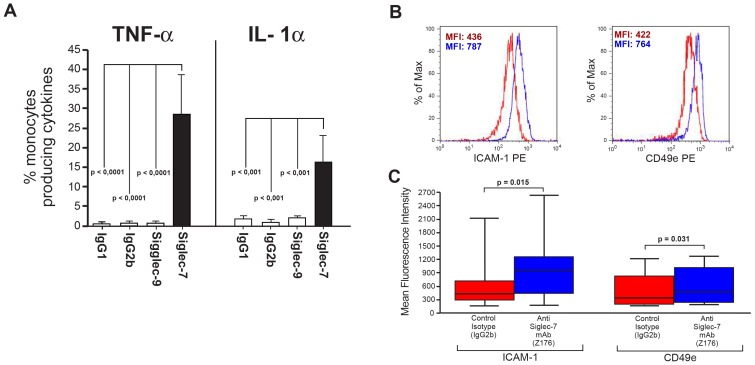
Intracellular production of TNF-α and IL-1α in monocytes upon engagement of Siglec-7 and Siglec-9 and modulation of adhesion molecules in monocytes upon engagement of Siglec-7. (A) Statistical histogram bar graph showing the percentages of CD14^pos^ monocyte producing TNF-α (left) and IL-1α (right) in the presence of mAbs cross-linking either Siglec-7 and Siglec-9 or their relative IgG2b and IgG1 isotype controls. Data are representative of 5 independent experiments performed in triplicates (± SD). (B) Representative flow cytometry histogram graphs showing the mean fluorescence intensity (MFI) of ICAM-1 (left) and CD49e (right) on CD14^pos^ monocyte after incubation with either the anti-Siglec-7 mAb (blue lines) or with the matched IgG2b isotype control (red lines). (C) Statistical summary graphs of box plots with medians and standard deviation showing the MFI of ICAM-1 (left) and CD49e (right) on CD14^pos^ monocyte after incubation with either the anti-Siglec-7 mAb (blue boxes) or with the matched IgG2b isotype control (red boxes). Data are representative of 5 independent experiments (± SD).

In order to confirm the real specificity of Siglec-7 in triggering the synthesis of pro-inflammatory cytokines and chemokines selectively in monocytes, we also cross-linked Siglec-9, another CD33-related lectin-type receptor that shares with Siglec-7 a similar structure and cellular distribution [1], [2]. We observed that the engagement of Siglec-9 with a specific anti-siglec-9 mAb, differently from Siglec-7, did not induce any significant production of IL-1α and TNF-α, measured as representative pro-inflammatory cytokines (Figure 3A). Finally, we triggered Siglec-7 function also on freshly isolated monocytes and also these experiments confirmed that the cross-linking of this lectin-type receptor on a pure population of monocytes induce or significantly increase the production of IL-6, IL-1α, MIP-1β, IL-8 and TNF-a if compared to isotype controls (Figure S2). The only differences we could observe is that the amount of the cytokines/chemokines produced upon engagement of Siglec-7 was lower in purified monocytes compared to those of total PBMCs, thus suggesting that cellular interactions might be relevant to provide stronger signals.

Since our data demonstrate that the ligation of Siglec-7 can elicit a monocyte-mediated inflammatory response and considering also that the migration of monocytes to inflamed tissue is dependent on adhesion to endothelia and extracellular matrix proteins [24], we tested whether the stimulation of Siglec-7 was also associated with an up-regulation of the following adhesion and co-stimulatory molecules in monocytes: CD11b, CD11c, CD18, CD49d, CD49e, ICAM-1, CD80 and CD86. Our results showed that the surface levels of the adhesion molecules ICAM-1 (CD54) and CD49e were significantly increased on monocytes incubated with the anti Siglec-7 mAb compared with those of monocytes incubated with isotype controls (Figure 3 B–C). In contrast, the expression of the other molecules tested was not affected by the engagement of Siglec-7 (data not shown).

### Phosphorylation of ERK is Associated with the Engagement of Siglec-7 in Monocytes

**Figure 4:**
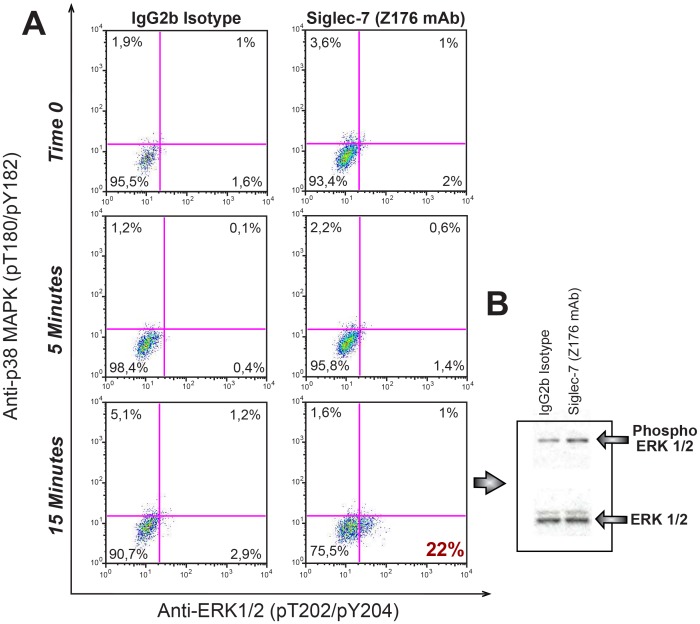
Phosphorylation of ERK upon engagement of Siglec-7 in monocytes. (A) Representative phospho-flow cytometry dot plot graphs showing the percentage of phosphorylation of p38 MAPK and of ERK 1–2 in freshly purified monocytes incubated with an IgG2b isotype (left column) and anti Siglec-7 mAb (right column) at time 0 (upper line) and after 5 (middle line) and 15 (lower line) minutes of incubation. The number highlighted in bold red within the lower right quadrant of the dot plot graph located in the lower line of right column indicates the phosphorylation of ERK following ligation of Siglec-7. (B) Representative western blot image showing the phoshorylation of ERK 1–2 in freshly purified monocytes stimulated with IgG2b isotype (left) and anti Siglec-7 mAb (right) after 15 minutes of incubation. Data are representative of 3 independent experiments (± SD).

The induction of an inflammatory response following the cross-linking of Siglec-7 is in agreement with another report showing that the treatment of human monocytes with an anti-CD33 mAb induced the Siglec-3-mediated production of IL-1, TNF-α and IL-8 [25]. Furthermore, several studies have recently pointed out that trans-membrane receptors containing ITIM may sometimes stimulate rather than repress cellular activation depending on the cellular context and that this “activating” ITIM cascade can be mediated by the extracellular signal-regulated kinases (ERK) pathway [26], [27], [28], [29], [30], [31], [32], [33]. Therefore, we tested whether or not the engagement of Siglec-7 in monocytes is associated with the phosphorylation of ERK. To this end, we incubated freshly purified monocytes either with the anti-Siglec-7 mAb or with the matched IgG2b isotype control in time-course experiments. We then analyzed the phoshorylation of ERK 1–2 by phospho-flow cytometry and compared it to phosphorylation given by the anti-p38 mitogen-activated protein kinases (MAPK), another important signaling pathway involved in the induction of many genes encoding inflammatory mediators [34]. Our data showed that the cross-linking of Siglec-7 was associated with detectable levels of phosphorylation of ERK 1–2 after 15 minutes of incubation, while it did not trigger the phosporylation of p38 MAPK. We could not detect any phosphorylation of ERK 1–2 and p-38 MAPK upon ligation the IgG2b matched isotype control (Figure 4). We then further validated the Siglec-7-induced phosporylation of ERK 1–2 by performing western blots. This additional experimental approach confirmed that the incubation of monocytes with the anti-Siglec-7 mAb for 15 minutes is indeed associated with an increased phosphorylation of ERK 1–2 as compared to that given by the matched isotype control. Indeed, although the detection of phosphorylated ERK 1–2 was expected even in monocyted treated with the IgG2b isotype control [35], the relative fold increase of ERK 1–2 phosphorylation in monocytes incubated with the anti-Siglec-7 mAb was of 32% higher than the one seen in monocytes stimulated with the IgG2b isotype control, calculated as an average of 3 independent experiments.

### Siglec-7 Contributes to the Establishment of Inflammatory Responses Following Recognition of Sialic Acid-free Pathogens

**Figure 5:**
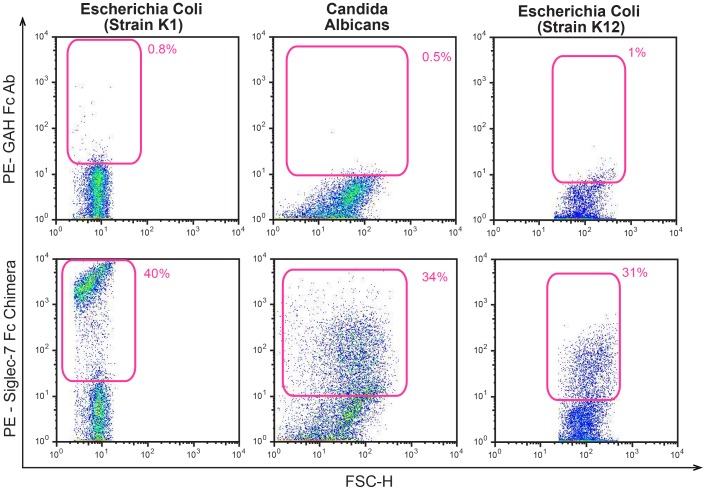
Binding of pathogens to Siglec-7 Fc chimera. Representative flow cytometric dot plot graphs showing the binding of goat anti human (GAH) Fc Ab (upper line) and of Siglec-7 Fc chimera (lower line) to *Escherichia coli* (strains K1 and K12) and *Candida albicans*. Data are representative of 3 independent experiments.

The fact that the cross-linking of Siglec-7, as well as of Siglec-3 [25], is associated with a monocyte-mediated pro-inflammatory response insinuates that a sialic-acid-independent alternative mechanism(s) of pathogen recognition might be possible. To validate this working hypothesis, we incubated different pathogens that either express or not express sialylated carbohydrates with a human Siglec-7 Fc chimera and we detected their ability to interact with the Siglec-7 fusion protein (Figure S3A). As expected, we observed that Siglec-7 is able to bind *Escherichia coli* (strain K1) and *Candida albicans*, two microbes known to express sialic acids [36], [37]. Surprisingly, we also found that Siglec-7 interacted with the strain K12 of *Escherichia Coli,* which has not been reported to express sialylated carbohydrates (Figure 5). We could not detect any interactions between the above-mentioned pathogens and goat anti-human Fc Abs alone, which were used as negative control.

**Figure 6:**
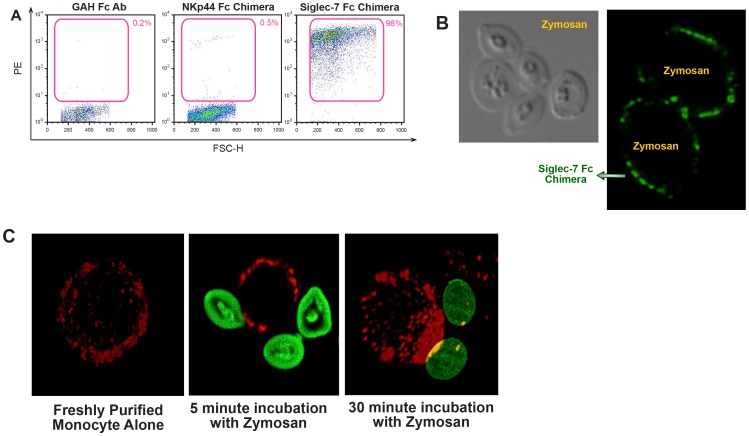
Binding of *Zymosan* to Siglec-7 Fc chimera and to Siglec-7 receptor expressed on freshly putified monocytes. (A) Representative flow cytometric dot plot graphs showing the binding of goat anti human (GAH) Fc Ab (left), NKp44 Fc chimera (middle) and Siglec-7 Fc chimera (right) to *Zymosan*. (B) Fluorescent microscopic images of *Zymosan* particles (gray, left part of the panel) surrounded by PE-labeled Siglec-7 Fc chimera (green, right part of the panel). (C) Fluorescent microscopic images of primary monocytes labeled for Siglec-7 (red) and incubated with *Zymosan* particles (green) for 5 and 30 minutes. The co-localization is labeled in yellow.

In order to confirm the real ability of Siglec-7 to bind microbes not expressing sialic acid residues and to investigate the functional correlates of these unexpected interactions, we analyzed the ability of Siglec-7 to bind *Zymosan*, a cell wall preparation derived from *Saccharomyces cerevisiae* that is widely used *in vitro* for the study of pathogen-host interactions [38]. Moreover, *Zymosan* yeast particles have not been reported to contain sialic acid residues and are well known for their ability to trigger the production of inflammatory cytokines and chemokines in immune cells through the binding of several *Zymosan* components (β-glucans, mannans, proteins, lipids) to different PRRs in human PBMCs and macrophages [39], [40], [41]. In line with what we observed with the acid sialic-free strain K12 of *Escherichia coli* (Figure 5), the Siglec-7 fusion protein was able to also bind *Zymosan*, while we could not detect any interactions of *Zymosan* with the goat anti-human Fc mAb alone or with NKp44 Fc chimera, another fusion protein used as a second negative internal control (Figure 6A–B). We then proceeded to detect the binding between Siglec-7 and *Zymosan* on freshly purified monocytes that constitutively express this receptor on their cell surface [1]. To this end, we incubated adherent monocytes with *Zymosan* and evaluated the localization of both Siglec-7 and the yeast particles on cell surface by confocal microscopy (Figure S3B). After 30 minutes of incubation there was a striking polarization of Siglec-7 distribution toward the pathogen and we also observed a co-localization of Siglec-7 and *Zymosan* on monocyte cell surface (Figure 6C).

**Figure 7:**
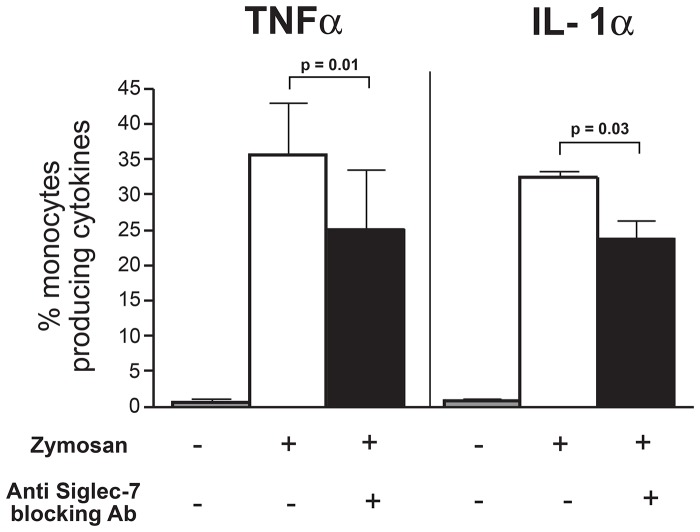
*Zymosan*-induced production of TNFα and IL-1α:masking of Siglec-7. Statistical histogram bar graph showing the percentages of CD14^pos^ monocyte producing TNF-α (left) and IL-1α (right) either in the absence (gray bars) or in the presence (white bars) of *Zymosan* and in presence of *Zymosan* cultured with blocking anti-Siglec-7 Abs (black bars). The intracellular production of TNF-α and IL-1α were evaluated by flow cytometric analysis. Data are representative of 5 independent experiments performed in triplicates (± SD).

**Figure 8:**
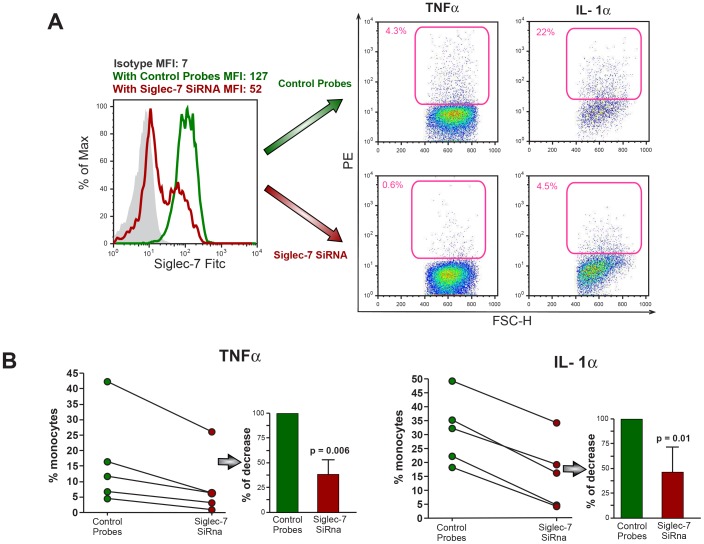
Zymosan-induced production of TNFα and IL-1α: silencing of Siglec-7. (A) Representative graphs showing the intracellular production of TNF-α and IL-1α in monocytes transfected either with non-targeting SiRNA control probes (green line in the histogram graph and dot plot graphs in the upper line of the panel) or with SiRNA duplexes specific for Siglec-7 (red line in the histogram graph and dot plot graphs in the lower line of the panel) in response to *Zymosan*. MFI: Mean Fluoresence Intensity. Filled gray Histogram: isotype control. (B) Summary graphs of dot plots (left) and statistical summary bar graphs with p values and standard deviation (right) showing the percentages of decrement of TNF-α (left) and IL-1α (right) in monocytes silenced for Siglec-7 compared to monocytes not silenced for Siglec-7 in response to *Zymosan*. Data are representative of 5 independent experiments (± SD).

We then proceeded to assess if Siglec-7 played any role in inducing a monocyte-mediated production of pro-inflammatory cytokines and chemokines following *Zymosan* recognition. To this end, we incubated freshly purified monocytes with these yeast particles in the presence or in the absence of polyclonal Abs neutralizing Siglec-7. Similar to previous reports [39], [40], [41], we observed that exposure of monocytes to *Zymosan* induced a remarkable production of both TNF-α and IL-1α. The masking of Siglec-7 with an anti-Siglec-7 Abs resulted in a statistically significant reduced production of TNF-α and IL-1α in monocytes (Figure 7). To further confirm the specific role of Siglec-7 in triggering an inflammatory response in primary monocytes upon binding with *Zymosan*, we performed mRNA silencing experiments. As shown in Figure 8A, we were able to markedly reduce the surface levels of Siglec-7 (up to 75%) in monocytes transfected with SiRNA duplexes specific for Siglec-7. We then exposed these monocytes silenced for Siglec-7 to *Zymosan*. This additional set of experiments confirmed that the levels of production of TNF-α and IL-1α were significantly lower in monocytes silenced for Siglec-7 compared to those of monocytes transfected with control non-targeting probes (scrambled SiRNA) (Fig. 6B). The same reduction was also obtained in regard to the production of IL-6, MIP-1β and IL-8 (data not shown). Taken together, these experimental results indicate that Siglec-7 actively participates in the enhancement of inflammatory responses by monocytes after pathogen recognition.

## Discussion

In the present study, we demonstrate that the engagement of Siglec-7 induces the production of TNF-α, IL-1α, IL-6, IL-8 and MIP-1β. The induction of this inflammatory response following ligation of Siglec-7 with a specific anti-Siglec-7 mAb is restricted to monocytes and does not occur in NK and T lymphocytes, the other two cell subsets constitutively expressing Siglec-7. This novel activating function of Siglec-7 is also corroborated by the fact that its cross-linking in monocytes leads to the phoshporylation of ERK 1–2, an important and well-established signaling pathway that activates a variety of transcription factors coordinating the modulation of many genes involved in the establishment of inflammatory responses [42]. We also show here that, other than binding pathogens expressing syalilated glycans, Siglec-7 can also interact with microbes not reported to be conjugated with sialic acid residues such as the strain K12 of *Escherichia coli* and *Zymosan* yeast particles. These findings provide novel mechanistic insights on the monocyte responses following the recognition of pathogens not expressing sialylated carbohydrates. Indeed, we also demonstrate here that masking or silencing Siglec-7 in monocytes incubated with *Zymosan* substantially reduced the production of the above-mentioned cytokines and chemokines, thus confirming that this CD33-related lectin-type receptor significantly contributes to prime an inflammatory outcome in monocytes.

The undetectable intracellular levels of the above-mentioned cytokines and chemokines after incubation with both IgG2a matched and IgG1 unmatched isotype negative controls indicate that the production of these pro-inflammatory molecules by monocytes is not associated with a non-specific binding between the Fc portion of mAbs and the CD16/FcγIII receptor, but is rather triggered by a selective cross-linking of Siglec-7. In this regard, PBMCs were also pre-incubated with human IgG antibodies that masked the CD16/FcγIII receptor expressed on monocytes and NK cells, thus avoiding non-specific interactions with the FC portion of mAbs. Indeed, the fact that we could not detect any NK cell-mediated synthesis of IL-6, IL-1α, MIP-1β, IL-8 and TNF-α either using anti-Siglec-7 mAb or isotype controls confirms one more that this phenomenon s associated with a selective engagement of Siglec-7 on monocytes and not to a non-specific stimulation of the CD16/FcγIII receptor.

The previously reported inhibitory function of Siglec-7 in regulating T cell receptor signaling and NK cell cytolytic activity reflects the structure of this ITIM-bearing receptor [1], [10], [21]. In this regard, it is widely accepted that the immune system employs negative regulation mechanisms of cellular responses through a large family of receptors containing ITIMs. Ligand engagement by these inhibitory receptors results in ITIM phosphorylation by Src family kinases, which, in turn, recruit phosphotyrosine phosphatase (PTP) such as Src homology region 2 domain-containing phosphatase-1 (SHP-1) and SHP-2. PTP recruitment switches off cellular pathways by decreasing tyrosine phosphorylation. Moreover, the engagement of inhibitory receptors containing ITIMs also prevents the activation signals that originate from receptors associated with immunoreceptor tyrosine based activating motifs (ITAMs) [43]. However, within the past few years, the interpretation of this antipathetic receptor-signaling paradigm has become less rigorous as the number of newly identified trans-membrane receptors containing ITIMs increases. Indeed, several studies recently reported that, in some circumstances and depending on the cellular context, ITIMs can propagate activation signals and ITAMs can mediate inhibition [26], [27], [28], [29], [30], [31], [32], [33]. This is the case of dendritic-cell-associated C-type lectin 2 (DCAL-2), an ITIM bearing receptor, whose ligation with an anti-DCAL-2 mAb recruits a SHP-2 domain that, instead of negatively regulating cell signaling, is involved in activating the ERK pathway and in inducing the production of IL-10, TNF-α, IL-6 and MIP-3β [26]. Many other ITIM-bearing receptors have been shown to recruit these “atypical” PTPs (both SHP-1 and -2) that partially explain the ambiguity of ITIM in immune-receptor signaling [28]. In line with these experimental evidences, we demonstrate here that the cross-linking of Siglec-7 by an anti-Siglec-7 mAb is also associated with the phosphorylation of the ERK pathway in monocytes. Hence, our results strongly suggest that the Siglec-7 isoform(s) expressed selectively on monocytes and not on T and NK cells belongs to the family of “activating” ITIM bearing receptors. Moreover, it has been shown that Siglec-7 inefficiently recruits PTP and that this phenomenon greatly affects the downstream signaling of this lectin-type receptor. In fact, introduction of a triple mutation into the Siglec-7 sequence greatly enhanced PTP recruitment and increased the inhibitory activity of the mutant close to that of Siglec-9 [44]. These experimental evidence indicate that the inhibitory potential of Siglec-7 is generally weaker compared to the one of other Siglecs or ITIM bearing receptors following cellular activation. Whether this weak recruitment of PTPs affects the inhibitory function of selected isoform(s) of Siglec-7 expressed on a particular cell subset or if this generally occurs in all immune cells constitutively expressing Siglec-7 is still not known. In any case, the weak engagement of PTP might be one of the reasons explaining the fact that Siglec-7, in contrast to other CD33-related receptors, does not induce apoptosis of leukemic cells while keeps the potential to inhibit their proliferation [45]. Both the weak recruitment of regular PTPs and/or the engagement of atypical activating forms of these phosphatases upon ligation of Siglec-7 in monocytes might explain the presence of a partial phosphorylation of the ERK pathway, as observed in our experiments. Therefore, our functional characterization of an activating form of a Siglec-7 containing ITIM domains follows the most recent updates in this research field, although deciphering the complex scenario of the immune-biology of ITIM and ITAM bearing receptors certainly represents an important challenge for better understanding the physiology and physiopathology of immune-receptor signaling.

The present study also reports that cross-linking of Siglec-7 on monocytes induced an up-modulation of two important adhesion molecules: ICAM-1 and CD49e. Interestingly, interactions between ICAM-1 with either β2 integrins lymphocyte function-associated antigen (LFA-1) or Macrophage 1 antigen (Mac-1) on endothelial cells induce transmigration of leukocytes during inflammatory responses. Moreover, ICAM-1 interaction with LFA-1 can deliver a co-stimulatory signal to T cells [46]. CD49e is an adhesion molecule that associates with CD29 to form integrin VLA-5, a fibronectin receptor [47]. Hence, the ligation of Siglec-7, other than inducing a monocyte-mediated inflammatory response, might also be important in regulating the migration of leukocytes toward sites of inflammation and in delivering co-stimulatory signals. The activation and the migration of monocytes to tissues lead to a plastic polarization toward M1 or M2 macrophages that depends from a variety of signals within the local micro-environment [48]. Although Siglec-7 is also expressed on macrophages (data not shown), it remains to be determined whether the engagement of this lectin-type receptors still trigger activating signals in macrophages or interfere with macrophage plasticity to undergo classic or alternative polarization.

Interest in Siglecs has grown remarkably in the recent few years as it has become evident that these receptors, broadly expressed on cells within the innate immune compartment and mostly mediating inhibitory signals upon ligation with sialylated carbohydrates, play a wide range of roles. Originally, the first reported function of Siglecs was to dampen host immune responses and set appropriate activation thresholds for regulating cellular growth, survival and the production of soluble mediators [1], [2]. Subsequently, it also became clear that, during the course of evolution, several pathogenic microorganisms have learned to synthesize or capture sialic acids in order to bind Siglecs and escape immune responses. Based on this, a novel and emerging working hypothesis postulates that CD33-related Siglecs also evolved in response to pathogen manipulation by associating their intracellular domains with either activating ITIMs, as previously described, or even with ITAM domains containing the adaptor molecule DAP12, as it has been described for Siglec-16 [8]. Our results are in line with this last experimental evidence by demonstrating that also Siglec-7 evolved to counterpart the gained ability of pathogens to synthesize or capture sialic acids in order to bind Siglec receptors and escape immune responses. To this end, Siglec-7 learnt to trigger activating signals in monocytes and to bind also sialic-free pathogens with the aim of providing optimal immune and inflammatory responses against pathogens. Blocking this function would theoretically give back to pathogens the possibility to evade innate immune responses. In this regards, Siglec-7 has already been reported to bind sialylated glycans either expressed by *Campylobacter jejuni* or acquired by *Pseudomonas aeruginosa* [14], [49]. Here, we confirm that Siglec-7 is indeed able to bind other pathogens expressing sialylated glycans such as strain K1 of *Escherichia coli* and *Candida albicans* [36], [37], but we also demonstrate the capacity of this CD33-related lectin-type receptor to interact with microbes not reported to express sialic acids such as the strain K12 of *Escherichia coli* and *Zymosan* yeast particles. In particular, our data showed that both Siglec-7 fusion protein and Siglec-7 receptor expressed on freshly purified monocytes bind *Zymosan*. Moreover, confocal images revealed that incubation of adherent monocytes with *Zymosan* induced a clear polarization of Siglec-7 towards the pathogen that co-localized with this lectin-type receptor at the binding site, thus supporting the role of Siglec-7 in pathogen recognition.

Our flow cytometry experiments also displayed that that the intensity of the binding (evaluated by mean fluorescence intensities) of Siglec-7 and the strain K12 *of Escherichia coli* is different compared to that of *Zymosan*, thus suggesting a different interaction affinity. Further studies are needed to disclose the nature and the kinetics of these sialic-acid independent bindings in order to disclose the Siglec-7 epitope(s) able to binds several and different pathogens. The scope of the present study is to assess whether, in the absence of the already reported sialic acid-mediated repressor activity of CD33 and CD33-related siglec molecules on immune cells [1], [2], [8], [25], Siglec-7 participates in the development of inflammation following pathogen recognition. This working hypothesis relies on the two above-reported experimental evidence: i) the ability of Siglec-7, as well as of CD33 [25], to trigger the production of pro-inflammatory cytochines and chemokines when cross-linked with a specific anti-siglec-7 mAb and ii) its capacity to bind sialic-acid-free pathogens. In fact, our data demonstrates that the incubation of monocytes with *Zymosan,* a sialic acid-free microbe widely used *in vitro* host-pathogen interactions [38], is indeed associated with a massive production of TNF-α and IL-1α. However, it is well known that the recognition of *Zymosan* by monocytes/macrophages occurs primarily through the engagement of different PRRs, including Dectin 1 and TLR2, two proteins that synergize in the induction of inflammatory responses [40]. Nevertheless, masking or silencing Siglec-7 in primary monocytes incubated with *Zymosan* greatly reduced the production of pro-inflammatory cytokines, thus demonstrating a clear contribution of Siglec-7 in triggering inflammation following the recognition of pathogens not expressing syalilated glycans. The fact that the production of pro-inflammatory mediators was not completely abrogated after blocking or silencing Siglec-7 confirms once again that different PRRs are required to trigger an inflammatory response in monocytes and that Siglec-7 is one of the actors playing an active role in this scenario. The synergy of Siglec-7 with other PRRs in triggering the activation of monocytes in response to pathogens remains to be determined. In this context, an important factor to consider is that the threshold for glycan recognition is a function of the density of membrane-bound and this greatly affects pathogen recognition and, as a consequence, the antagonistic or synergic activation of signaling pathways [50]. Hence, additional studies are required to address the kinetic and the interaction of Siglec-7 with other PRRs in monocytes, that differently from the other two lymphocyte subsets constitutively expressing Siglec-7 (i.e. NK and T cells), are innate effectors designated to bind and phagocitate microbes, and to subsequently induce inflammatory responses.

In conclusion, the present study demonstrates a novel activating role of Siglec-7 in monocytes but not in T and NK cells, the other two immune cell subsets constitutively expressing this CD33-related lectin-type receptor. Indeed, although the glycobiology of Siglec-7 (as well as of Siglecs in general) has been extensively investigated over the past decade, only a few studies disclosed the functional immune correlates of this receptor and none of these reports have analyzed Siglec-7 functions within the monocyte compartment. We show here that Siglec-7 ligation with an anti-Siglec-7 mAb is able to induce an inflammatory outcome selectively in monocytes. The activating role of Siglec-7 also occurs when sialic acid-free *Zymosan* yeast particles binds this lectin-type molecule on monocytes. This indicates that the engagement of Siglec-7 contributes in developing inflammation in response to pathogenic microorganisms not expressing syalilated glycans at least in certain cellular contexts. Our findings open new perspectives in the field by disclosing a new function of the Siglec-7 innate immune receptor following pathogen recognition.

## Materials and Methods

### Isolation of PBMCs and Monocytes

Human PBMCs were obtained from buffy coats and healthy volunteers signed consent forms in accordance with clinical protocols approved by the Institutional Review Board of Desio Hospital, Milan, Italy. The percentage of monocytes within the white cell compartment of all healthy donors tested is comprised within the normal range of 10% and no one of the donors showed high titers of serum antibodies in any of their sub-classes (IgM, IgG, sIgA). PBMCs were isolated over Ficoll-Hypaque gradient centrifugation (Amersham Pharmacia Biotech) and CD14^pos^ and/or CD16^pos^ monocytes were purified from PBMCs by negative magnetic cell sorting technique (EasySep Human Monocyte Enrichment Kit, Stemcell Technologies) according to the protocol provided by the manifacturer. This experimental approach allowed us to isolate all CD3^neg^, CD19^neg^, CD56^neg^ monocytes subsets either single o double positive for CD14 and CD16 [51].

### Protein Array

96 flat-bottom well plates were coated with 10 µg/ml of anti-Siglec-7 mAb (clone Z176, IgG2b) (either purchased from Beckman Coulter or provided by Dr. Alessandro Moretta) or with the same amounts of either a matched IgG2b or an unmatched IgG1 isotype controls (Beckman Coulter). After 16 hours of incubation, coated plates were washed twice with PBS medium (Gibco) supplemented with 2% FCS (Hyclone). 2×10^6^/ml of PBMCs were suspended in 1641 RPMI medium supplemented with 10% FCS with penicillin/streptomycin and L-glutamine (Gibco) and cultured for 18 hours at 37°C in the previously described coated plates at the final volume of 200 ml/well. Before culturing the cells in the plates coated with anti-Siglec-7 mAb, PBMCs were pre-incubated with human IgGs antibodies (Sigma-Aldrich) at 4°C for 20 minutes to block CD16/FcγIII receptor and to avoid its non-specific bindings with the FC portion of mAbs. Cell supernatants were then collected and analyzed using the RayBio Biotin Label-based Human Antibody Array that allows the detection of 507 different proteins, following manufacturer’s instructions (RayBio® Biotin Label-based Human Antibody Array I, RayBiotech, Inc.).

### Flow Cytometry and Confocal Microscopy

2×10^6^/ml of PBMCs or freshly purified monocytes were incubated as described above in 96 flat-bottom plates coated with anti-Siglec-7 and anti-Siglec-9 (Biolegend, IgG1) mAbs or with isotype controls in the presence of monesin (Golgi-Stop, 0,3 µl/ml) (BD Pharmigen). For multicolour flow cytometric analyses (FACS Canto II, BD Biosciences), cells were stained with phycoerythrin-Cy5 labelled CD56 (Beckman & Coulter), phycoerythrin-Cy7 labelled CD3, allophycocyanin-Cy7 labelled CD19, fluorescein isothiocyanate labelled CD14 (BD Biosciences).

The surface levels of adhesion and co-stimulatory molecules were detected by using phycoerythrin-labelled anti-CD11b, anti- CD11c, anti-CD18, anti-CD49d, anti-CD49e, anti- ICAM-1, CD80 and anti- CD86 mAbs (BD Pharmigen).

For intracellular staining, after an incubation of 30 minutes at 4°C with the above mentioned anti-CD56, -CD3, -CD-19 and -CD14 labelled mAbs, PBMCs were washed, fixed and permeabilized with cytofix/cytoperm following manufacturer’s instructions (BD biosciences) and incubated separately for 30 minutes at 4°C with the following Phycoerythrin labelled anti-cytokine/chemokine mAbs: anti-IL-6, anti-IL-1α, anti-IL-1β, anti-MIP-1β, anti-IL-8, anti-TNF-α and anti IFN-γ (BD biosciences). Data were analyzed using FlowJo software (TreeStar).

For binding experiments, *Zymosan* (Zymosan A, Bioparticles, Invitrogen), *Escherichia coli* K-1 (ATCC) and K12 strains (Molecular probes) and *Candida albicans* (Greer Laboratories) were incubated for 30 minutes at 37°C with human Siglec-7 and NKp44-Fc chimeras (R&D Systems) in PBS medium. Pathogens were then washed twice in PBS and anti-human FC antibodies labelled with Phycoerythrin (for flow cytometry) or with Alexa 488 (for confocal microscopy) (Invitrogen) were added to the culture (Figure 4A). After incubating for 30 minutes at 4°C, samples were washed and analyzed. For confocal microscopy, the samples were first fixed with paraformaldehyde (PFA) at 4% and the binding between fusion proteins and *Zymosan* was then detected with an Olympus Fluoview FV1000 laser-scanning confocal microscope. Images (1024×1024 pixels) were acquired with a 60×1.4 NA Plan-Apochromat oil immersion objective (Olympus, Hamburg, Germany). Images were processed using Leica TCS-NT/SP software (version 1.6.587), Imaris 3.3.2 (Bitplane AG), and Adobe and Photoshop 7.0 (Adobe systems).

For detecting the binding between Siglec-7 receptor and *Zymosan* particles on freshly purified monocyte, 250,000 adherent monocytes were incubated in PBS medium with 1×10^4^ particle of *Zymosan* directly conjugated with Alexa 488 (Invitrogen). Cells were then washed and stained with anti-Siglec 7 mAb (Z176) followed by Alexa 633 conjugated isotype-specific rat anti-mouse second Ab (Invitrogen). After washing and fixing with PFA at 4% cells were cyto-spinned and mounted on glass slides with a prolonged antifade reagent (ProLong® Gold Antifade Reagent, Invitrogen). Images were acquired and processed immediately, after 5, 30 and 60 minutes of incubaton with Zymosan (Supplemental Fig. 1).

### Phospho-flow Cytometry and Western Blot Analysis

For phospho-flow cytometric analyses, 2×10^5^/well of freshly purified monocytes were cultured in complete medium and incubated in time course experiments (0, 5 and 15 minutes) at 37°C in 96 flat bottom plates coated either with an anti-Siglec-7 mAbs or with a matched IgG2b isotype control. Cells were then fixed with BD Cytofix Buffer, incubated for 10 minutes at 37°C, washed twice with PBS-2% FCS and permeabilized with BD Phosflow Perm Buffer III for 30 minutes on ice. After washing, cells were incubated for 30′ at RT with anti-pERK 1–2 Alexa 488 and anti-p38 PE antibodies.

For Western Blot Analysis, 2×10^5^/well of freshly purified monocytes were incubated at 37°C in time course experiments (0, 5 and 15 minutes) with anti-Siglec-7 mAbs or with a matched IgG2b isotype control in serum free-medium. Cells were then lysed in a radio-immune-precipitation (RIPA) buffer (50 mM TrisHCl, pH 8, 1% TritonX100, 100 mM NaCl, 1 mM MgCl_2_) supplemented with Complete Mini Protease Inhibitor Cocktail (Roche Diagnostics, Indianapolis, IN) and with 200 mM Sodium fluoride, 10 mM Sodium pyrophosphate, 100 mM Sodium orthovanadate. Following incubation on ice for 30 minutes, cell lysate was centrifuged at 16000 g for 30 minutes at 4°C, boiled for 5 minutes in Leammli buffer, inserted in a 10% SDS-polyacrylamide gel electrophoresis (SDS-PAGE) and transferred to nitrocellulose membranes. Immunoblotting was performed using primary rabbit polyclonal antibody specific for phospho-ERK (p44/42; Thr202/Tyr204), detected with an antirabbit HRP-conjugated Ab and visualized using chemiluminescent substrate (Millipore, Bedford, MA, USA). Membranes were then stripped with hot Stripping buffer (62,5 mM Tris-HCl, pH 6,8, 100 mM β-mercaptoethanol, 2% Sodium Dodecyl Sulphate) and re-probed with rabbit polyclonal antibodies that detected total ERK 1–2. All antibodies were purchased from Cell Signaling Technology (Beverly, MA). Radiographic images were quantified by scanning densitometry and expressed as percent of relative fold increase. All reagents used for phospho-flow and western blot experiments were endotoxin-free.

### Masking Experiments

Freshly purified monocytes were incubated for 30 minutes at 37°C with 100 mg/ml of *Zymosan* either in the presence or the absence of a pre-incubation (1 hour at 4°C) with 10 mg/ml of a blocking anti-Siglec-7 polyclonal antibody (R&D system). Cells were washed twice, and the intracellular production of TNF-α and IL1-α was detected as previously described.

### RNA Interference Assay

Siglec-7 silencing on freshly purified monocytes was performed using *Stealth Select RNAi™ siRNA technology* following manufacturer’s instructions (Invitrogen). Briefly, two different pre-designed Siglec-7 siRNA probes provided by Invitrogen (5′-GAACUUGACUGUGACGUCUUCCAA-3′ and 5′-GAACAGUGUGUGUCAUUCUCUCCAA-3′) were annealed with complementary sequences and successively transfected into monocytes using Lipofectamin 2000 (Invitrogen). A different predesigned SiRNA probe, provided by Invitrogen, was used as internal control (5′-GAGAUACGGGUAGAAUAGCGACAAA-3′). Cells were incubated at 37°C in complete RPMI medium supplemented with GM-CSF at 10 ng/ul (Schering-Plough). Surface expression of Siglec-7 on monocyte was detected after 3 days of incubation by flow cytometry using the anti Siglec-7 mAb (Z-176) followed by fluorescein isothiocyanate conjugated isotype-specific goat anti-mouse second reagent (Southern Biotechnology Associates). The intracellular production of TNF-α and IL1-α following *Zymosan* incubation was detected as previously described.

### Statistical Analyses

Immune response distributions within monocyte populations were evaluated using non parametric Wilcoxon signed rank tests. All p values are 2-sided and unadjusted.

## Supporting Information

Figure S1 **Gating strategy gating within total freshly purified PBMCs and intracellular production of IL-6 upon engagement of Siglec-7.** (A) Within the lymphocyte gate, T cells were identified on the basis of their CD3 expression, while the CD3^neg^/CD19^neg^/CD56^pos^ phenotype characterized NK cells. The positivity for CD14 distinguished primary monocytes within their distinctive gate. (B) Representative flow cytometry dot plot graphs showing the percentage of CD3^pos^ T cells (first column), CD56^pos^ NK cells and CD14^pos^ monocytes producing IL-6 in response to either the anti-Siglec-7 mAb (lower line) or to the matched IgG2b isotype control (upper line). (C) Summary graph of dot plots with medians (horizontal black bars) showing the percentage of freshly purified NK cells, monocytes and CD8^pos^ T cells constitutively expressing Siglec-7 receptor on their surface.
(TIF)

Figure S2 **Intracellular production of pro-inflammatory cytokines and chemokines in purified monocytes upon engagement of Siglec-7.** Statistical summary graphs of dot plots with medians (horizontal black bars) and p values showing the percentage of freshly purified CD14^pos^ monocytes producing IL-6, IL-1α, MIP-1β, IL-8 and TNF-α in response to either the anti-Siglec-7 mAb (red circles) or the matched IgG2b isotype control (green circles).
(TIF)

Figure S3 **Methodology detecting the binding between Siglec-7 and pathogens.** (A) Siglec-7 fusion protein was incubated with pathogens (*Escherichia coli, Candida albicans)* or *Zymosan* yeast particles and the related binding was detected trough a goat anti human (GAH) Fc Ab labeled with Phycoerythrin (flow cytometry) or with Alexa Fluor 488 (confocal microscopy only for experiments with *Zymosan*). (B) Adherent monocytes stained with anti human Siglec-7 mAb directly conjugated with Alexa Fluor 633 were incubated with *Zymosan* particles directly conjugated with Alexa Fluor 488.
(TIF)
