# Supplementary figures and images for: Engagement of Siglec-7 Receptor Induces a Pro-Inflammatory Response Selectively in Monocytes
Source: PLoS One. 2012 Sep 28;7(9):e45821. doi: 10.1371/journal.pone.0045821 (PMC3461047; doi:10.1371/journal.pone.0045821)

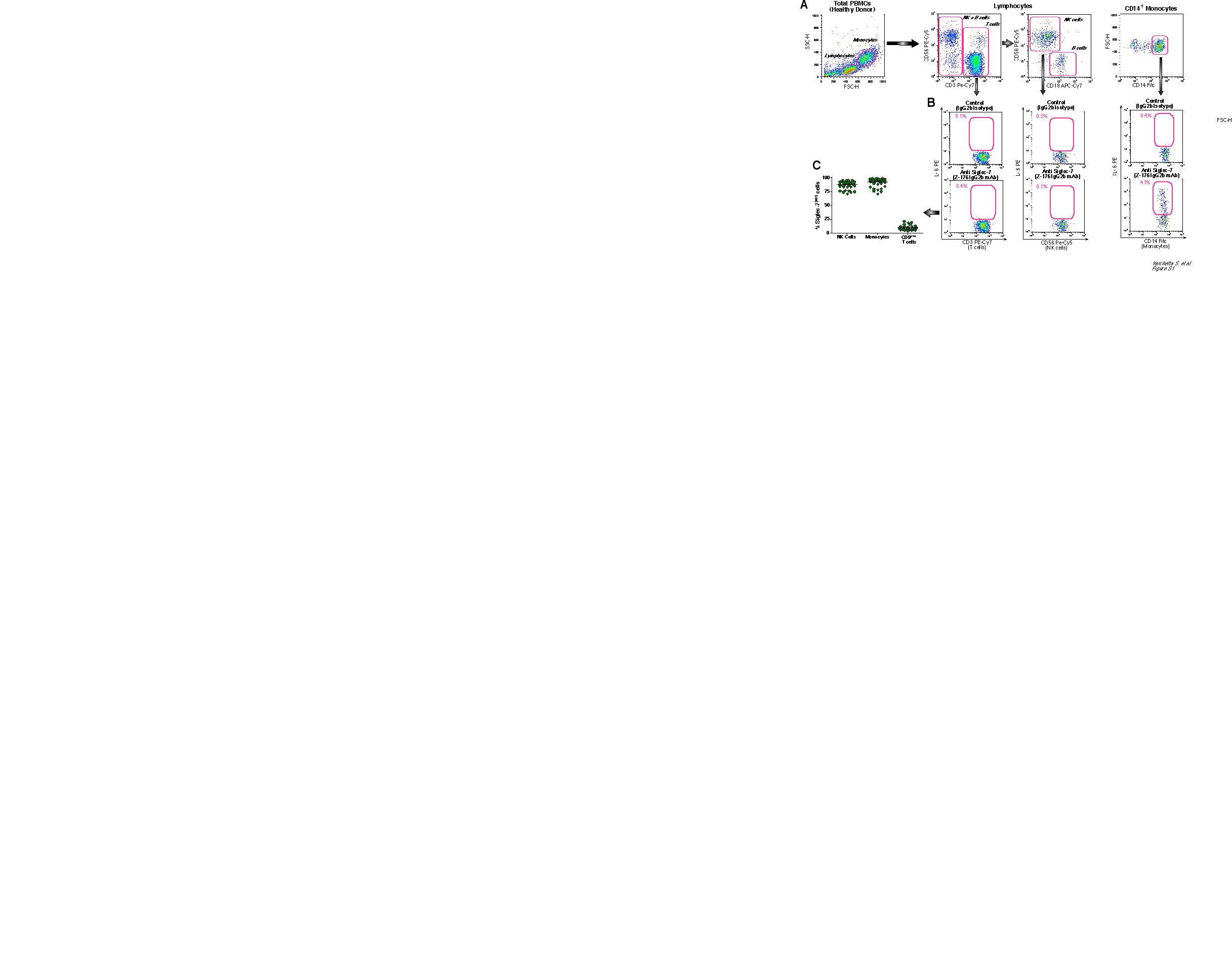

Supplement: Figure S1 — Gating strategy gating within total freshly purified PBMCs and intracellular production of IL-6 upon engagement of Siglec-7. (A) Within the lymphocyte gate, T cells were identified on the basis of their CD3 expression, while the CD3neg/CD19neg/CD56pos phenotype characterized NK cells. The positivity for CD14 distinguished primary monocytes within their distinctive gate. (B) Representative flow cytometry dot plot graphs showing the percentage of CD3pos T cells (first column), CD56pos NK cells and CD14pos monocytes producing IL-6 in response to either the anti-Siglec-7 mAb (lower line) or to the matched IgG2b isotype control (upper line). (C) Summary graph of dot plots with medians (horizontal black bars) showing the percentage of freshly purified NK cells, monocytes and CD8pos T cells constitutively expressing Siglec-7 receptor on their surface. (TIF) [file pone.0045821.s001.tif]

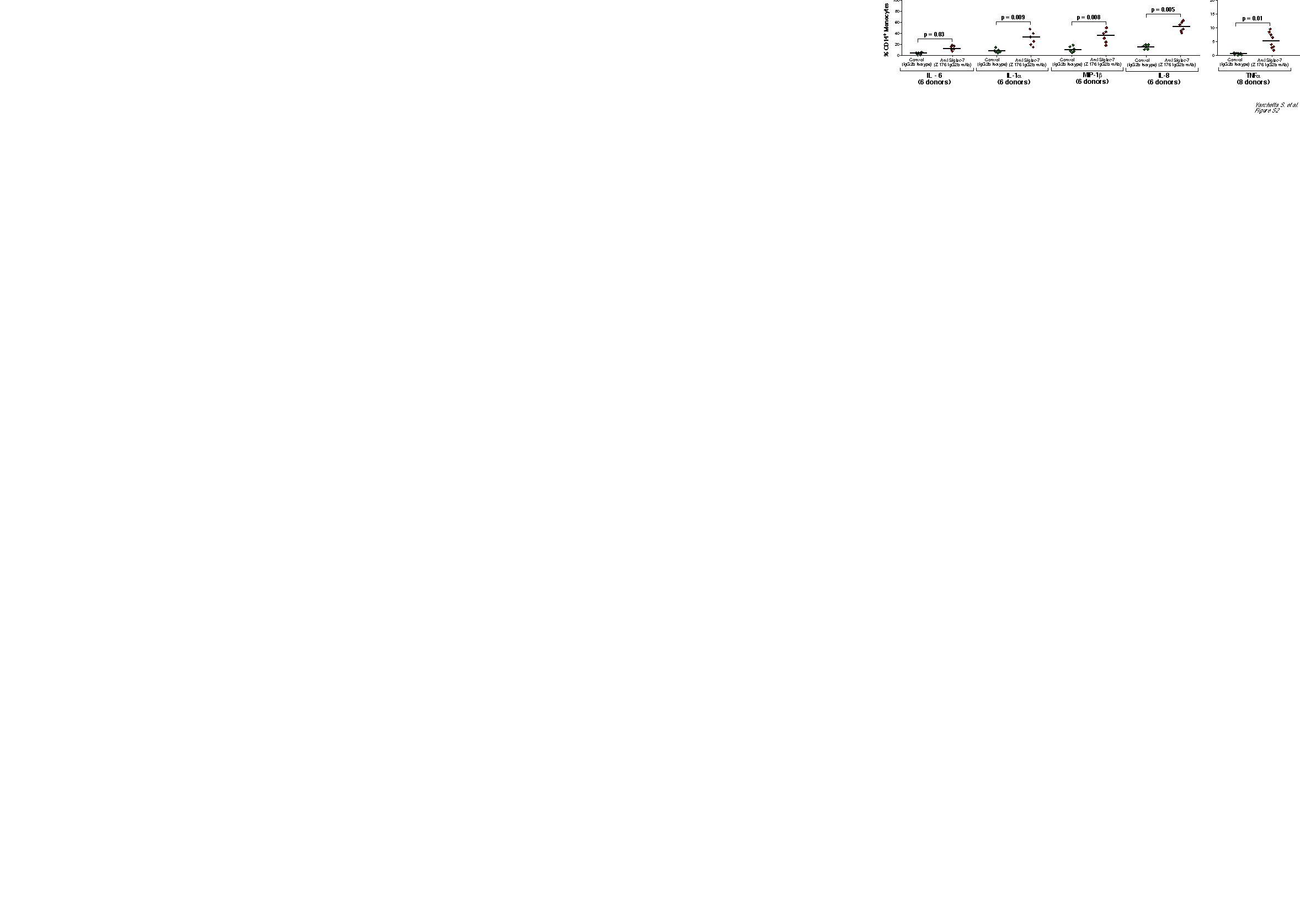

Supplement: Figure S2 — Intracellular production of pro-inflammatory cytokines and chemokines in purified monocytes upon engagement of Siglec-7. Statistical summary graphs of dot plots with medians (horizontal black bars) and p values showing the percentage of freshly purified CD14pos monocytes producing IL-6, IL-1α, MIP-1β, IL-8 and TNF-α in response to either the anti-Siglec-7 mAb (red circles) or the matched IgG2b isotype control (green circles). (TIF) [file pone.0045821.s002.tif]

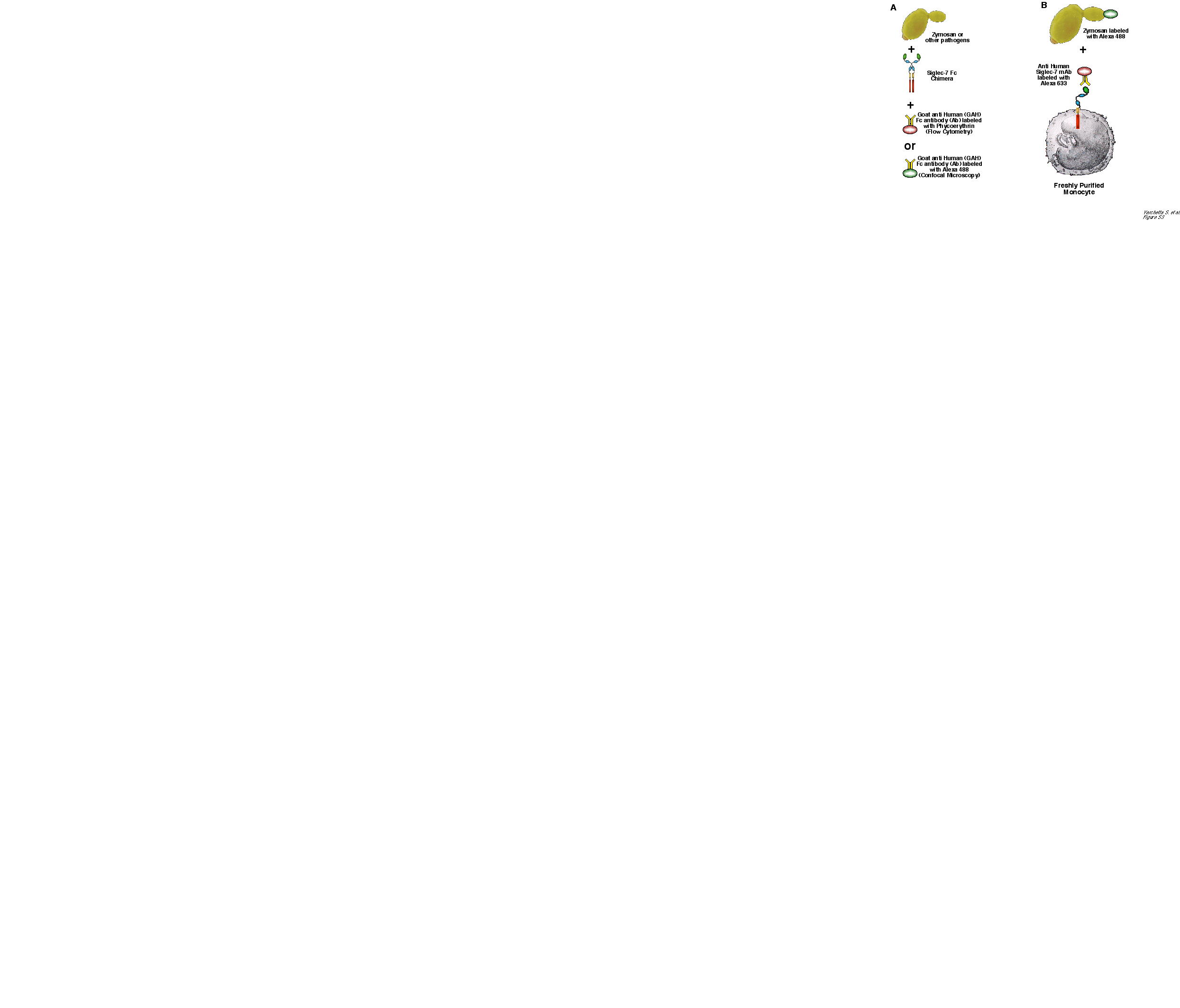

Supplement: Figure S3 — Methodology detecting the binding between Siglec-7 and pathogens. (A) Siglec-7 fusion protein was incubated with pathogens (Escherichia coli, Candida albicans) or Zymosan yeast particles and the related binding was detected trough a goat anti human (GAH) Fc Ab labeled with Phycoerythrin (flow cytometry) or with Alexa Fluor 488 (confocal microscopy only for experiments with Zymosan). (B) Adherent monocytes stained with anti human Siglec-7 mAb directly conjugated with Alexa Fluor 633 were incubated with Zymosan particles directly conjugated with Alexa Fluor 488. (TIF) [file pone.0045821.s003.tif]
